# Association between Tryptophan Metabolism and Inflammatory Biomarkers in Dairy Cows with Ketosis

**DOI:** 10.3390/metabo13030333

**Published:** 2023-02-23

**Authors:** Zhengzhong Luo, Kang Yong, Zhenlong Du, Yixin Huang, Tao Zhou, Li Ma, Xueping Yao, Liuhong Shen, Shumin Yu, Zuoting Yan, Suizhong Cao

**Affiliations:** 1 Department of Clinical Veterinary Medicine, College of Veterinary Medicine, Sichuan Agricultural University, Chengdu 611130, China; 2 Department of Animal Science and Technology, Chongqing Three Gorges Vocational College, Chongqing 404100, China; 3 Lanzhou Institute of Animal Husbandry and Veterinary Pharmaceutical, Chinese Academy of Agricultural Sciences, Lanzhou 730050, China

**Keywords:** ketosis, dairy cows, inflammatory biomarkers, tryptophan metabolism

## Abstract

Dairy cows with ketosis have high circulating beta-hydroxybutyric acid (BHBA) concentrations alongside which inflammation is concomitantly developed. Tryptophan (Trp) is an essential amino acid that participates in the regulation of the inflammatory response. However, the association between Trp metabolism and inflammation in dairy cows with ketosis remains unclear. Therefore, blood samples from healthy (*n* = 10) and ketotic (*n* = 10) primiparous dairy cows were collected at the calving date and the day of ketosis diagnosis (7 days in milk (7 DIM)). Serum levels of non-esterified fatty acids (NEFA), BHBA, haptoglobin (HP), serum amyloid A (SAA), lipopolysaccharide, and cortisol were analyzed. Tryptophan and its metabolites were quantified using liquid chromatography–tandem mass spectrometry. At 7 DIM, the concentrations of NEFA, BHBA, HP, and SAA were higher and the levels of Trp, kynurenine (KYN), indoleacetic acid, indole-3-lactic acid, and 3-indoxyl sulfate were lower in the dairy cows with ketosis compared with those in the healthy cows. However, the KYN/Trp and melatonin/Trp ratios increased in the cows with ketosis. At the calving date, the serum lipopolysaccharide levels did not differ between the healthy and ketotic cows, whereas the levels of NEFA, HP, and cortisol increased in the ketotic cows. Correlation analysis showed that Trp deficiency and elevated Trp metabolism in the dairy cows occurred during ketosis. Overall, our results suggest that abnormal Trp metabolism may contribute to the pathogenesis of ketosis.

## 1. Introduction

Dairy cows experience a metabolic challenge during the transition period, especially after calving, characterized by an unbalanced energy status [1,2]. To adapt to this negative energy balance, dairy cows mobilize body fat, which is accompanied by elevated circulating concentrations of non-esterified fatty acids (NEFA) that are common in cows during the post-partum period [3,4]. Poor metabolic adaptation can result in a higher concentration of NEFA, which increases the risk of metabolic disease after calving [5,6]. Some researchers have suggested that NEFA contribute to the development of ketosis and displaced abomasum [5,7,8]. Traditionally, beta-hydroxybutyric acid (BHBA) is generated from the NEFA in the liver via partial oxidation and then released into certain organs, such as the heart and brain, as an alternate fuel source [9]. However, when NEFA produce excessive BHBA in response to poor metabolic adaptation, dairy cows’ blood BHBA concentration increases to more than 1.2 mmol/L and ketosis occurs [10,11]. During the early lactation period, particularly during the first week of lactation, 40–60% of dairy cows suffer from ketosis [11,12,13]. The prevalence of subclinical ketosis is higher than that of clinical ketosis in lactating dairy herds [14,15]. Evaluations of economic loss indicate that ketosis is not the main factor affecting milk production but it increases the risk of other diseases and culling [16,17]. Sordillo et al. [18] and Pascottini et al. [19] have reported significant negative effects caused by the inflammatory response to ketosis. The levels of inflammatory biomarkers, such as haptoglobin (HP), serum amyloid A (SAA), and lipopolysaccharide (LPS), increase in dairy cows with ketosis [20,21,22].

Tryptophan (Trp) is an essential aromatic amino acid whose metabolites participate in the regulation of inflammation and insulin resistance [23,24]. Tryptophan metabolism follows three major pathways: (1) Trp is converted into kynurenine (KYN) in the liver and immune tissue, which is mediated by the two rate-limiting enzymes tryptophan 2,3-dioxygenase (TDO) and indolamine-2,3-dioxygenase (IDO) [25]. (2) Serotonin or 5-hydroxytryptamine is generated from Trp in enterochromaffin cells via hydroxylation. The serotonin is acetylated to form N-acetylserotonin (NAS) in order to finally produce melatonin (MLT) [26]. Finally, (3) Trp is directly transformed into several molecules, such as indole-3-lactic acid (ILA), indoleacetic acid (IAA), and indole, by the gastrointestinal microbiota [27]. In cows, besides BHBA and NEFA, KYN was recently reported as a new indicator of negative energy balance [28]. The levels of Trp and KYN in blood samples are lower in dairy cows with ketosis than in healthy cows [29]. In addition, serotonin is involved in the regulation of metabolic homeostasis in dairy cows during transition periods [30]. However, changes in Trp metabolism in dairy cows from calving to lactation have rarely been studied, and the association between Trp metabolism and inflammation in ketosis events remains unclear.

Therefore, in this study, we aimed to determine the role of Trp metabolism in ketosis development. The concentrations of plasma Trp metabolites in dairy cows with ketosis were quantified using liquid chromatography–tandem mass spectrometry (LC-MS/MS). The levels of inflammatory biomarkers in the serum were concurrently analyzed on the calving date and the day when ketosis was diagnosed. Individual Trp metabolites and serum variables were integrated to assess the potential functional links between the different Trp metabolism pathways and inflammatory responses. Our findings provide novel information regarding the pathogenesis of ketosis in dairy cows.

## 2. Materials and Methods

### 2.1. Animals and Study Design

The experimental protocol was approved by the Institutional Animal Care and Use Committee of Sichuan Agricultural University (NO. DYY-2018203039). The study was conducted at a modern dairy farm in Sichuan Province, China. In this study, 90 primiparous, pregnant dairy cows of similar age and number of days into gestation were selected and housed in cow barns. The prepartum dairy cows were fed twice daily with a total mixed ration consisting of 29.04% corn silage, 32.67% oat hay, 7.26% wheat straw hay, 29.14% complete feed (Hengfeng Feed Co. LTD, Sichuan, Meishan, China), 1.45% soybean meal, and 0.44% choline on a dry matter basis. Dairy cows were transferred to fresh barns after calving and were fed three times daily with a total mixed ration consisting of 18.80% corn silage, 17.27% alfalfa hay, 4.32% oat hay, 32.61% complete feed (Sichuan Hengfeng Feed Co. LTD, Meishan, China), 6.47% cottonseed, 6.48% beet pulp, 12.95% steam-flaked corn, and 1.10% molasses on a dry matter basis. The cows had free access to fresh water. The dry matter intake of the cows was recorded daily. Lactating cows were milked daily at 06:30, 14:00, and 21:30 in a 60-point rotary milking parlor (Afimilk, Kibbutz Afikim, Israel). Dairy cows’ health was monitored by vets. The evaluation criteria regarding health status, including clinical signs, rumen fill scores, body condition scores, and fluctuations in milk yield, were described as reported previously [31]. In addition, the evaluation criteria for diseases, such as milk fever, mastitis, metritis, retained placenta, abomasal displacement, clinical ketosis (BHBA ≥ 3.0 mmol/L), and diarrhea, were described in our previous report [32]. A blood BHBA concentration of ≥1.2 mmol/L in the first week post-calving was classified as ketosis, and subclinical ketosis was defined as 1.2 mmol/L ≤ BHBA < 3.0 mmol/L [10,11]. Before the morning feeding, blood samples were collected from all cows via the caudal vein at 1 day in milk (DIM; calving occurred within 6 h after the colostrum was released) and 7 DIM. Blood BHBA concentration was determined at 1 and 7 DIM using a Blood Ketone Meter (WD1621, Nova Bio Vet, Waltham, MA, USA). Serum and plasma (using heparin sodium as an anticoagulant) samples were collected, centrifuged at 1500× *g* for 10 min at 25 °C, and subsequently stored at −80 °C.

In this study, 20 healthy cows were monitored (mean ± standard error of the mean (SEM); BHBA = 0.64 ± 0.02 mmol/L at 1 DIM and BHBA = 0.91 ± 0.04 mmol/L at 7 DIM). Cows with diseases had said ailments diagnosed. There were 15 cows with only sub-clinical ketosis (BHBA = 0.69 ± 0.03 mmol/L at 1 DIM and BHBA = 2.29 ± 0.07 mmol/L at 7 DIM), while there were 55 with signs of other diseases, including 5 with milk fever, 4 with mastitis, 11 with metritis, 10 with retained placenta, 5 with abomasal displacement, 6 with clinical ketosis, 2 with diarrhea, and 12 with more than two diseases or others. Ten cows that were diagnosed with only sub-clinical ketosis were of a similar age and had similar female calf weights, body condition scores, calving ease scores, and actual days of pregnancy at last parity (Table 1). For comparison with the former animals, 10 healthy cows in similar condition as mentioned above were randomly selected to form a control group.

### 2.2. Serum Markers Analyses

Serum NEFA, HP, SAA, LPS, and cortisol levels were determined using commercially available test kits from Nanjing Jiancheng Bioengineering Institute, China. The levels of serum HP, SAA, LPS, and cortisol were analyzed using a competitive enzyme-linked immunosorbent assay (inter-assay coefficients of variability (CV) < 12% and intra-assay CV < 10%). NEFA concentration was determined using a coupled enzymatic reaction system (acyl CoA Synthetase-acyl CoA oxidase method, inter-assay CV < 8%, and intra-assay CV < 10%).

### 2.3. Quantification of Tryptophan and Its Metabolites

The deuterated internal standards MLT-d_4_ and creatinine-d_3_ (Sigma-Aldrich, Merck, St. Louis, MO, USA) were added to 200 μL aliquots of plasma samples and mixed with 800 μL of acetonitrile-methanol (1:1, *v*/*v*). Plasma samples were pretreated as reported previously [33]. In brief, all mixtures were sonicated in an ice water bath and incubated at −20 °C for 60 mi; thereafter, they were centrifuged at 14,000× *g* for 20 min at 4 °C. The supernatant was collected and vacuum-dried. A total of 100 μL acetonitrile:water (1:1, *v*/*v*) was added and mixed. The supernatant was collected by centrifugation at 14,000× *g* for 15 min at 4 °C. Subsequently, the supernatant was analyzed for Trp and its metabolites using liquid chromatography– (LC; 1290 Infinity II, Agilent Technologies, Santa Clara, CA, USA) tandem mass spectrometry (MS/MS; QTRAP 6500, AB SCIEX, Framingham, MA, USA). In LC analysis, the mobile phase consisted of A (5 mM ammonium acetate and 0.2% ammonium hydroxide in water) and B (acetonitrile with 0.5% ammonium hydroxide). The gradient elution procedure was as follows: 0–5 min, 5% to 60% B; 5–11 min, 60% to 100% B; 11–13 min, 100% B; 13–13.1 min, 100% to 5% B; and 13.1–16 min, 5% B. The MS was equipped with an electrospray ionization source and operated alternately in positive-ion mode with +4.5 kV and negative-ion mode with −4.5 kV ion spray voltage. The source temperature was maintained at 580 °C, and the curtain gas was supplied at 35 psi. Ion source gas 1 and gas 2 were provided at 45 psi and 60 psi, respectively. MS/MS data were collected with a multiple reaction monitor, and the peak area was acquired using the MultiQuant software (v3.0.2; AB SCIEX, Framingham, MA, USA). Tryptophan (CAS#73-22-3), KYN (CAS#2922-83-0), serotonin (CAS#50-67-9), NAS (CAS#1210-83-9), MLT (CAS#73-31-4), IAA (CAS#87-51-4), ILA (CAS#1821-52-9), and 3-indoxyl sulfate (IS; CAS#487-94-5) (Sigma-Aldrich, Merck, MO, USA) were used as internal standards for the quantification of targeted metabolites in the plasma. The calibration curve and corresponding regression coefficients were obtained based on the concentration gradient of the standard, and the targeted metabolite concentrations were subsequently calculated.

### 2.4. Statistical Analysis

The chemical structures of the targeted metabolites were generated using the ChemDraw 20.0 software (PerkinElmer Informatics, Waltham, MA, USA). To compare the variables between the healthy and ketotic groups, a two-tailed Student’s *t*-test was performed using the SPSS software (v21.0, IBM, Armonk, NY, USA). Repeated measures ANOVA was also performed. The DIM scores were defined as fixed effects in the model, and each cow was defined as a random effect. The threshold of significance was set at *p* < 0.05, and trends toward significance were declared at 0.05 ≤ *p* < 0.10. Graphics were generated using the GraphPad Prism software (v9.0, GraphPad, San Diego, CA, USA). Data are expressed as mean ± SEM. The associations between Trp metabolism and clinical parameters were determined using Spearman’s rank correlation in the R platform (v4.2, https://www.r-project.org, accessed on 15 December 2022). Correlation thresholds were set to Spearman’s *r* > 0.4 or *r* < –0.4 and *p* < 0.05.

## 3. Results

### 3.1. Alteration of Milk Yield, Dry Matter Intake, and Serum Markers in the Healthy and Ketotic Cows

**Figure 1:**
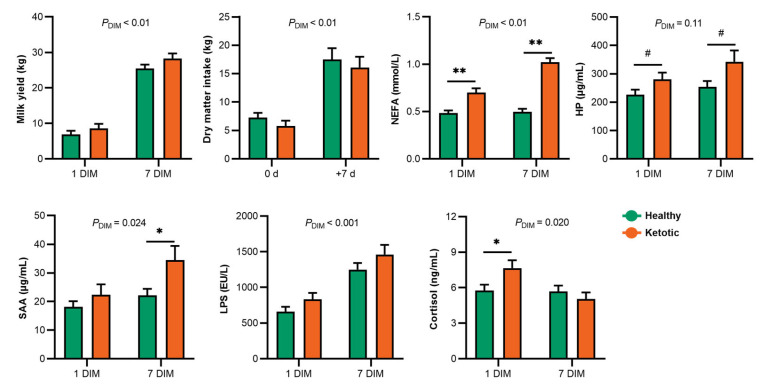
Bar plot indicating the changes in dry matter intake, milk yield, non-esterified fatty acids (NEFA), haptoglobin (HP), serum amyloid A (SAA), lipopolysaccharide (LPS), and cortisol between the healthy and ketotic cows at 1 and 7 days in milk (DIM). 0.05 ≤ ^#^ *p* < 0.1, * *p* < 0.05, and ** *p* < 0.01.

There was no significant difference regarding the dry matter intake and milk yield in the dairy cows between the healthy and ketotic groups at 1 DIM and 7 DIM (Figure 1). At 7 DIM, the mean milk yields in the healthy and ketotic cows were 25.45 kg and 28.26 kg, respectively. At 7 DIM, the blood NEFA concentrations in the ketotic cows were significantly higher (*p* < 0.01) than those in the healthy cows. Noticeably, at 1 DIM, the NEFA concentration in the ketotic cows was significantly higher (*p* < 0.01) than that in the healthy cows. Compared with those in the healthy cows at 1 DIM, the serum HP and cortisol levels increased (*p* < 0.1) in ketotic cows. However, SAA and LPS levels did not differ between the healthy and ketotic cows at 1 DIM. At 7 DIM, the SAA and HP levels increased (*p* < 0.1) during the ketosis event. Serum SAA and LPS levels in the dairy cows increased significantly (*P*_DIM_ < 0.05) from 1 to 7 DIM.

### 3.2. Alteration in Tryptophan Metabolism

**Figure 2:**
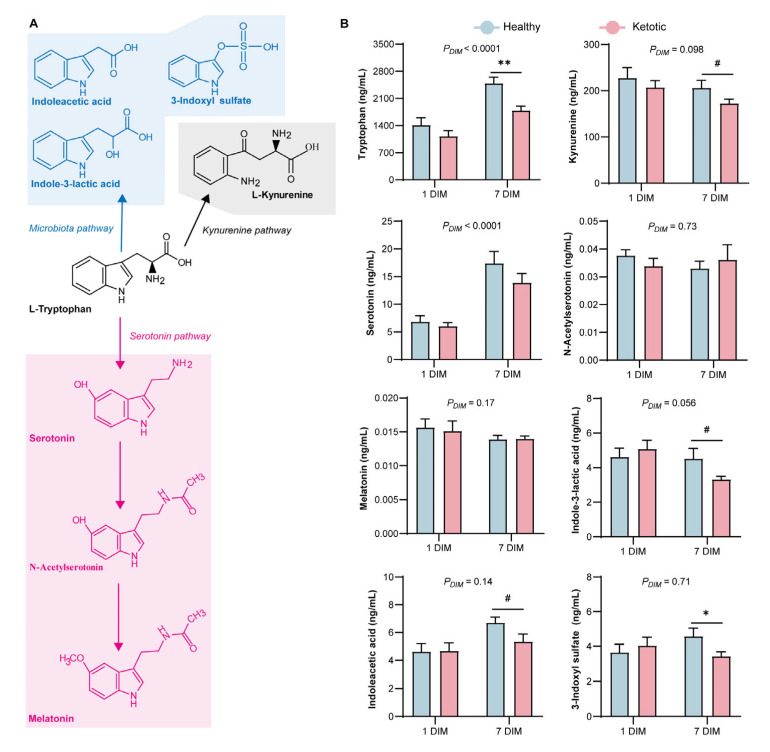
Tryptophan (Trp) metabolism in dairy cows during the ketosis event. (**A**) Diagram of Trp metabolism pathway, including kynurenine, serotonin, and microbiota pathways. (**B**) Levels of Trp and its main metabolites between the healthy and ketotic groups at 1 and 7 days in milk (DIM). 0.05 ≤ ^#^ *p* < 0.1, * *p* < 0.05, and ** *p* < 0.01.

**Figure 3:**
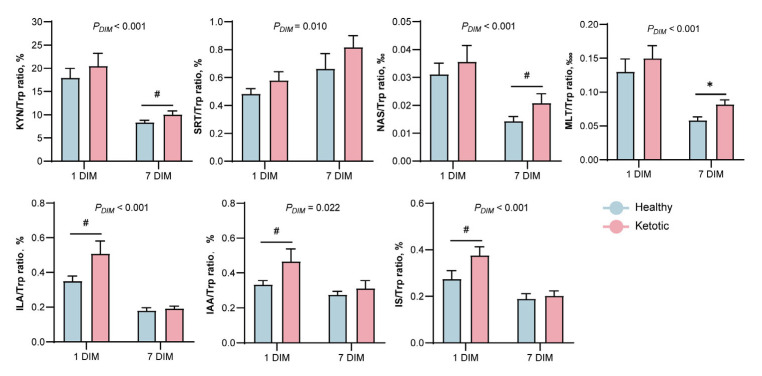
Ratios of individual metabolites to Trp between the healthy and ketotic dairy cows. Trp, tryptophan; KYN, kynurenine; SRT, serotonin; NAS, N-acetylserotonin; MLT, melatonin; ILA, indole-3-lactic acid; IAA, indoleacetic acid; IS, 3-indoxyl sulfate. 0.05 ≤ ^#^ *p* < 0.1, * *p* < 0.05.

Tryptophan metabolism consists of KYN, serotonin, and microbiota pathways, which were altered during the ketosis event (Figure 2A). The plasma Trp and IS concentrations were significantly lower (*p* < 0.05) in the ketotic group than those in the healthy group at 7 DIM (Figure 2B). In contrast to those in the healthy cows, the concentrations of KYN, ILA, and IAA showed a decreasing trend (0.05 < *p* < 0.1) in the ketotic cows. The levels of eight metabolites in the Trp metabolic pathway, namely, Trp, KYN, serotonin, NAS, MLT, IAA, ILA, and IS, did not differ between the healthy and ketotic groups at 1 DIM. However, the ILA/Trp, IAA/Trp, and IS/Trp ratios were higher (*p* < 0.1) in the ketotic group than those in the healthy group at 1 DIM (Figure 3). Additionally, the KYN/Trp, NAS/Trp, and MLT/Trp ratios were higher (*p* < 0.1) in the ketotic group than those in the healthy group at 7 DIM.

### 3.3. Association between Tryptophan Metabolism and Inflammatory Biomarkers during Ketosis Events

**Figure 4:**
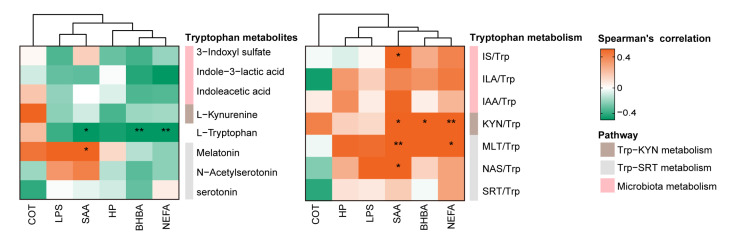
Tryptophan and its metabolites associated with clinical phenotypes during the ketosis events. NEFA, non-esterified fatty acids; BHBA, beta-hydroxybutyric acid; SAA, serum amyloid A; HP, haptoglobin; LPS, lipopolysaccharide; COT, cortisol; Trp, tryptophan; KYN, kynurenine; SRT, serotonin; NAS, N-acetylserotonin; MLT, melatonin; ILA, indole-3-lactic acid; IAA, indoleacetic acid; IS, 3-indoxyl sulfate. * *p* < 0.05, ** *p* < 0.01.

To explain the associations between Trp metabolism and inflammatory challenges during the ketosis events, Trp-related metabolites were integrated with serum biomarkers based on Spearman’s correlation analysis (Figure 4). The decrease in Trp concentration was negatively correlated with increased levels of NEFA (*r* = −0.71, *p* < 0.001), SAA (*r* = −0.54, *p* = 0.015), and BHBA (*r* = −0.60, *p* = 0.006) levels. During the ketosis events, an increased KYN/Trp ratio was positively correlated with the increased NEFA (*r* = 0.62, *p* = 0.004), BHBA (*r* = 0.55, *p* = 0.012), and SAA (*r* = 0.53, *p* = 0.018) levels. In addition, the IS/Trp ratio was positively correlated with the SAA level (*r* = 0.57, *p* = 0.01) during ketosis events. Notably, the SAA level was positively correlated with the NAS/Trp (*r* = 0.49, *p* = 0.029) and MLT/Trp (*r* = 0.58, *p* = 0.008) ratios. The circulating NEFA level was positively correlated with the MLT/Trp ratio (*r* = 0.54, *p* = 0.012).

## 4. Discussion

Dairy cows with ketosis present with metabolic stress, which is characterized by excessive lipid mobilization and inflammatory dysfunction [18,20]. The dietary supplementation of dairy cows with tryptophan has been recommended to relieve stress and improve production during the lactation period [34]. Via a metabolomics approach, Trp metabolites have been found to participate in the development of ketosis [28,29]. However, the mechanisms underlying Trp metabolism and ketosis remain unclear. In this study, we used a targeted metabolomics method to analyze the levels of Trp and its metabolites in dairy cows on the calving date and the day on which ketosis was diagnosed and concurrently integrated them with inflammatory biomarkers. Our results demonstrate that inflammatory biomarker levels increased and Trp metabolism was enhanced in the dairy cows with ketosis. Notably, the ratio of Trp metabolites was altered in the ketotic cows during calving, indicating that abnormal Trp metabolism may be associated with inflammation before ketosis is confirmed. Our findings provide novel mechanistic insights into the pathogenesis of ketosis.

Dairy cows experience excessive lipid mobilization even before the confirmation of ketosis [35,36]. In the present study, we found that the circulating NEFA concentration was markedly higher in the ketotic cows, but the BHBA levels did not differ on the calving date, which is consistent with the results of Ha et al. [37] and Turk et al. [38]. In general, NEFA metabolism has two harmful pathways: (1) abundant NEFA reassemble to produce triglyceride (TG) in the hepatocellular endoplasmic reticulum, which further causes TG accumulation in the liver; and (2) carnitine palmitoyltransferase 1 transports NEFA to the mitochondria, where ketone bodies are subsequently generated from NEFA metabolism through incomplete oxidation [2,4]. TG storage likely precedes BHBA synthesis in dairy cows after calving [39,40]. Notably, NEFA play an important role in the development of inflammatory responses in periparturient dairy cows [41]. Palmitic acid, a Toll-like receptor-4 agonist, is a major component of NEFA and induces a pro-inflammatory response by activating the NF-κB pathway [42]. Dairy cows with hyperketonemia exhibited higher palmitic acid levels [29,32]. Acute-phase proteins, including HP and SAA, are regarded as biomarkers for evaluating the inflammation statuses of dairy cows [43]. NEFA concentration was strongly positively correlated with HP and SAA levels in dairy cows during the transition period [44]. In line with our findings, few studies have reported that the HP and SAA levels increased during ketosis events [20,22]. Furthermore, cortisol and LPS are important induction factors that promote the expression of acute-phase proteins in hepatocytes [45]. In the present study, LPS levels did not differ between the healthy and ketotic cows from the calving date to 7 DIM, but the serum cortisol concentration increased at 1 DIM. Abuajamieh et al. [20] found that the circulating LPS level in ketotic dairy cows did not alter post-calving, whereas ketotic cows suffered from the challenge of a high concentration of LPS before the prepartum period. Pascottini et al. [19] also suggested that metabolic stress and systemic inflammation in dairy cows during the prepartum period may result in the development of ketosis after calving. Therefore, the dysregulation of inflammation is an important contributor to the development of ketosis, but the factors that lead to differences in systemic inflammation warrant further investigation.

Tryptophan is an essential amino acid produced in the diet and plays a crucial role in the regulation of immune function [46]. Notably, Trp deficiency occurs in dairy cows with hyperketonemia [29,42,47]. In addition, the Trp levels in dairy cows decreased during the ketosis event, and the levels of partial metabolites, including KYN, ILA, IAA, and IS, also decreased in the present study. However, a decrease in Trp levels may lead to lower concentrations of the downstream metabolites. Therefore, the ratio of individual downstream metabolites to Trp was analyzed to explain the changes in Trp metabolism during the ketosis events. In the present study, KYN and serotonin pathways were enhanced in the ketotic dairy cows on the day of diagnosis. The KYN pathway is a major route of Trp metabolism and is regulated by IDO and TDO [25,48]. The expression of IDO and TDO is induced by proinflammatory cytokines [49,50] and corticosteroids [51], respectively. Larsson et al. [52] demonstrated that the plasma Trp level decreased and KYN/Trp ratio increased following LPS treatment. Furthermore, the KYN/Trp ratio was positively correlated with cortisol levels with respect to the cows’ inflammatory statuses [53]. Importantly, KYN is metabolized by enzymatic oxidation and non-enzymatic cyclization to quinolinic acid and further to nicotinamide adenine dinucleotide (NAD^+^) via the Preiss–Handler pathway [54]. KYN-derived NAD^+^ regulates the programming of the inflammatory response by modulating the level of succinate (a tricarboxylic acid cycle metabolite) and maintaining redox homeostasis [55].

In contrast, serotonin is converted into NAS by arylalkylamine N-acetyltransferase and further metabolized to MLT by hydroxyindole-O-methyl transferase [56]. MLT relieves palmitic acid-induced cytotoxicity by attenuating oxidative and endoplasmic reticulum stress [57]. In addition, MLT lowers the expression of acute-phase proteins, including SAA, HP, and C-reactive protein, in bovine mammary epithelial cells stimulated with LPS and plays a key role in anti-inflammation [58]. However, Horst et al. [59] suggested that circulating serotonin was not correlated with NEFA and BHBA levels in ketotic dairy cows, which is consistent with the results of the present study. Furthermore, microbial Trp catabolites participate in inflammatory regulation and the development of metabolic disease [60,61,62]. For example, *Bifidobacterium adolescentis*, *Bacteroides fragilis*, *Bacteroides thetaiotaomicron*, and *Eubacterium cylindroides* in the intestine convert Trp into ILA and IAA [27,63]. Krishnan et al. [60] and Ehrlich et al. [64] reported that IAA and ILA attenuate pro-inflammatory cytokine expression via the activation of the aryl hydrocarbon receptor. Unexpectedly, Trp-derived indole is transferred into the liver and further converted to IS by cytochrome P450 2E1 and sulfotransferases [65]. IS is cytotoxic at high concentrations and enhances the expression of interleukin-6 and SAA [66]. Likewise, we found that the IS/Trp ratio was positively correlated with SAA levels during the ketosis events. Our study also found that IAA/Trp, ILA/Trp, and IS/Trp ratios increased in the ketotic group cows at their respective calving dates. Thus, Trp metabolism may participate in the progression of inflammation in dairy cows with ketosis through feedback regulation, which requires further research.

## 5. Conclusions

Dairy cows experience excessive lipid mobilization and inflammation before a diagnosis of ketosis is made. We found that the Trp catabolism of gastrointestinal microbiota in the ketotic dairy cows was enhanced on their respective calving dates. Despite the circulating Trp deficit in cows during ketosis events, Trp metabolism was elevated and closely correlated with inflammatory biomarkers. This study suggests that elevated tryptophan metabolism may be a consequence of ketosis. Future studies are required to analyze the regulatory enzymes involved in Trp metabolism and the downstream metabolites of KYN, which may provide new therapeutic and preventative options.

## Figures and Tables

**Table 1:** Calf weight, calving ease score, body condition score, age, actual days of pregnancy, and beta-hydroxybutyric acid level between the healthy and sub-clinical ketotic dairy cows.

Item	Healthy Cows (*n* = 10)	Sub-Clinical Ketotic Cows (*n* = 10)
Calf weight (kg), female	37.90 ± 0.84	38.20 ± 0.97
Calving ease score	1.10 ± 0.09	1.20 ± 0.13
Age (month) ^1^	27.34 ± 1.06	28.08 ± 0.94
Day of pregnancy (d)	275.40 ± 1.06	274.50 ± 0.47
Body condition score ^2^	3.50 ± 0.04	3.60 ± 0.06
BHBA concentration at 1 DIM	0.63 ± 0.03	0.72 ± 0.04
BHBA concentration at 7 DIM	0.89 ± 0.05	2.36 ± 0.09 **

^1^ The age of dairy cows on the seventh day after calving. ^2^ Body condition score was assessed on the seventh day before the due date. DIM = days in milk. Data are expressed as mean ± SEM. ** *p* < 0.01.

## Data Availability

Data is not publicly available due to privacy or ethical restrictions.
